# Late massive radial artery pseudoaneurysm following cardiac catheterization: A case report

**DOI:** 10.1016/j.ijscr.2021.105774

**Published:** 2021-03-16

**Authors:** Radhika Sharma, Pujan Patel, John N. Catanzaro

**Affiliations:** Division of Cardiology, Department of Internal Medicine, University of Florida, 655 W 8th St., 5th Floor, Ambulatory Care Center, Jacksonville, FL, 32209, United States

**Keywords:** Coronary angiography, Radial artery pseudoanuerysm, Cardiac catheterization complications

## Abstract

**Introduction:**

Transradial artery approach for cardiac catheterization was first introduced in the late 1980s and has now become the approach of choice due to its anatomical advantage, reduction in complications, and overall improved patient experience.

**Case presentation:**

We present a case of a 77 year-old female who presented with an extremely rare and late complication of radial artery pseudoaneurysm after transradial coronary intervention. The patient presented at a post-procedural follow-up with severe pain at the radial access site and was found to have a partially thrombosed pseudoaneurysm. Given the anatomical variance of the pseudoaneurysm, the patient underwent successful open surgical repair.

**Clinical discussion:**

Although radial artery access is a relatively safe approach in comparison to the transfemoral approach, the risk of adverse events still exists. Among the complications of the transradial approach, pseudoaneurysms are relatively rare, occurring in less than 0.1% of cases. Regardless, early identification of this complication is essential to timely intervention.

**Conclusion:**

Our case highlights the importance of post procedural monitoring and early identification and diagnosis of the complication to facilitate appropriate therapy.

## Highlights

• Radial artery access for cardiac catherization can have complications, rarely a pseudoanuerysm can form.
• Imaging modalities to diagnose a pseudoaneursym including arterial ultrasound can computed tomography can help with diagnosis.
• Management of a pseudoaneurysm depends on the severity and can involve compression techniques, thrombin injections, or surgical repair.

## 1 Introduction

Cardiac catheterization is the gold standard in defining coronary anatomy and intervening on critical lesions. Majority of complications have been related to arterial access site, previously done via the femoral artery. With the introduction of arterial access via the radial artery, the overall complications due to access site issues have dramatically decreased [1,3]. Though radial artery access is a relatively safe approach, the risk of adverse events still exists. Commonly reported complications of the radial artery approach include radial artery spasm, non-occlusive arterial injury, forearm hematoma, and asymptomatic arterial occlusion [5]. Rare complications include symptomatic arterial occlusion, pseudoaneurysm, and artery perforation [5,6]. We present a case with a rare complication of a radial artery pseudoaneurysm after a cardiac catheterization.

## 2 Case presentation

**Fig. 1:**
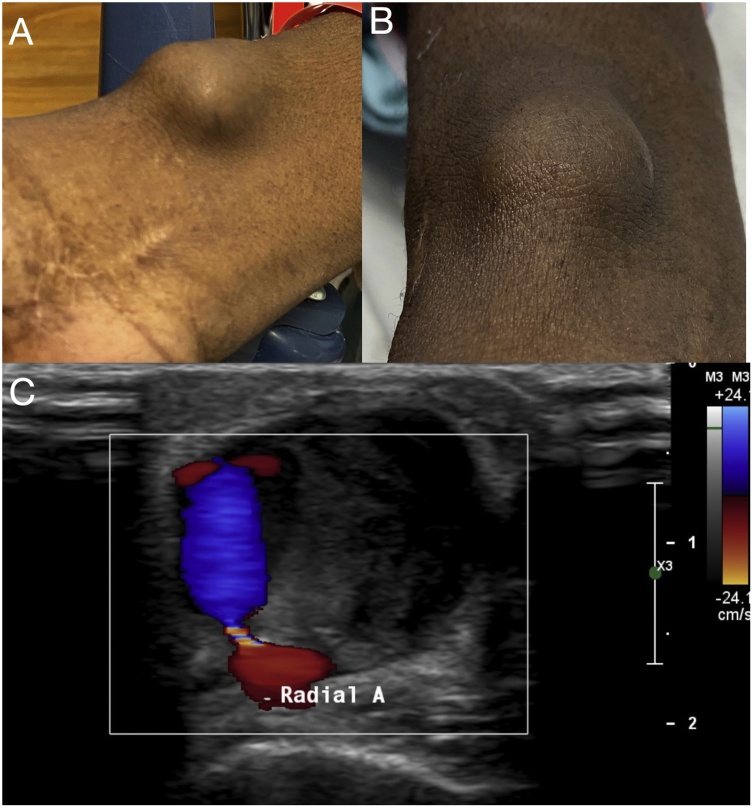
**A & B**: Visual inspection of radial aetery pseudoaneurysm. **C**: Duplex Ultrasound of radial artery with partially thrombosed, large pseudoaneurysm measuring at 3.2 × 1.8 cm and a 1.3 cm neck.

A 77 year old female with a history of atrial fibrillation on apixaban, systolic heart failure, and chronic stable angina. She presented to her cardiologist’s office with compliants of worsening chest discomfort for the past two months. She reported compliance with her medical therapy which included the antiaginal medications metoprolol tartrate, amlodipine and isosorbide dinitrate. For evalauation of her worsening angina, she underwent a cardiac catheterization. Right radial artery access was obtained using a 6 french sheath. Coronary angiography demonstrated nonobstructive coronary artery disease. Standard sheath removal protocol involving a radial artery compression device band was applied over the access site with successful hemostasis. She presented at the four-month clinic follow-up with severe pain at the radial access site. A large, nonmobile, tender mass was visualized without ecchymosis and full range of motion (Fig. 1A and B). Duplex ultrasonography revealed a 3.2 × 1.8 cm partially thrombosed pseudoaneurysm with turbulence at the neck measuring 1.3 mm (Fig. 1C). Given the small caliber of the neck, the pseudoaneurysm was not amenable to endovascular repair. However due to persistent symptoms, the patient underwent successful open surgical repair.

## 3 Discussion

Cardiac catheterization is a common procedure done to evaluate coronary anatomy and to intervene on critical lesions via angioplasty and stenting. The frequency of catheterizations has increased since its introduction in the 1970’s and now, more than a million procedures are done annually [3]. For catheterizations that require an arterial access site, the common femoral and radial artery are the two common sites of percutaneous approach. Historically the femoral artery was the initial access site, however with the advent and introduction of the transradial approach, there has been a reduction in access site complications and mortality, a decrease in length of stay, and an overall enhancement of patient comfort [3,4]. The transradial approach has not only increased in prevalence in comparison to the transfemoral approach, it has now become the approach of choice [9].

Although the transradial approach has a lower incidence of major access-related complications and is safer than the transfemoral approach, periprocedural complications have been documented. The most common complication is asymptomatic radial artery occlusion, which due to the collateral arterial supply of the hand, only rarely results in adverse events [5,9]. Other commonly reported complications of the radial artery approach include radial artery spasm, non-occlusive arterial injury, and forearm hematoma. Rare complications include symptomatic arterial occlusion, pseudoaneurysm, and radial artery perforation [6].

As in the case described, radial artery pseudoaneurysms are relatively rare, occurring in less than 0.1% of cases [2,8]. Pseudoaneurysms occur with defective healing of the catheterization puncture site. Failure in healing of the vessel allows for a continuous loss of arterial blood and further development of a hematoma with connecting neck and turbulent blood flow [4,8]. The presentation may consist of a palpable, pulsatile mass, progressive enlargement, pain, and an audible bruit [2]. Once suspected, further evaluation can be performed via imaging, with arterial ultrasound as the initial imaging modality. Pulsatility, turbulent internal flow, and a hematoma with variable echogenicity are specific signs of radial pseudoaneurysm [5]. Pathognomonic for a pseudoaneurysm is the “ying-yang” or “to-and-fro” sign which is best seen with color flow doppler and represents the variation of flow through the neck between systole and diastole [2,7]. Additional imaging such as computed tomography can help to delineate the site of the pseudoaneurysm in cases of discrepant findings or for further definition to assist with surgical planning.

Once diagnosed, further management depends on the severity of the pseudoaneurysm. Spontaneous thrombosis is possible if the pseudoaneurysm is smaller than 3 cm, has a long and narrow neck, and low flow volume [3]. Mechanical and/or pharmacologic treatment modalities can be further considered if spontaneous thrombosis does not occur or with increased severity of the complication. Initial management begins with application of ultrasound-guided continuous pressure proximal to the site of the pseudoaneurysm [2,4,7]. This can be done either manually or with a TR band, the goal being that continuous pressure will ultimately cause obliteration of the pseudoaneurysm neck. Continuous pressure treatment modalities may fail for large pseudoaneurysms with a wide neck and in patients on systemic anticoagulation and antiplatelet therapy [7]. If pressure does not result in thrombosis of the pseudoaneurysm, a percutaneous thrombin injection should be considered. Although thrombin injections provide faster results than continuous pressure (6 s compared to 45 min for the latter), they are associated with risk of arterial thrombosis, embolization, and limb ischemia [2]. Ultrasound-guided compression and thrombin injection are non-operative treatment modalities for pseudoaneurysms with a long, narrow neck [7]. Open repair or surgery is considered after failure of conservative treatment or for pseudoaneurysms with difficult morphologic features, predominantly short and wide necks. The most common procedures include hematoma evacuation, arterial repair, and stalk excisions, with arterial ligation reserved for emergency situations involving arterial rupture or limb ischemia.

Although pseudoaneurysms are relatively rare and occur in less than .1% of all transradial catheterization cases, it is important to use proper technique and instrumentation to prevent access site complications.

Avoidance of multiple puncture attempts, use of smaller sheaths, adequate anticoagulation, and use of appropriate post-procedural compression devices may reduce the risk of complications [4].

## 4 Conclusion

Radial artery pseudoaneurysms are a rare complication of cardiac catheterization done via radial artery access. Our case highlights the importance of post procedural monitoring and early identification and diagnosis of the complication to facilitate appropriate therapy.

## SCARE criteria

The work has been reported in line with SCARE 2020 criteria per the SCARE 2020 guideline [10].

## Declaration of Competing Interest

The authors report no declarations of interest.

## Funding

None.

## Ethical approval

The following case report is exempt from ethical approval.

## Consent

Written informed consent was obtained from the patient for publication of this case report and accompanying images. A copy of the written consent is available for review by the Editor-in-Chief of this journal on request.

## Author contribution

Each author contributed equally in the manuscript preparation and review of literature.

## Registration of research studies

Not Applicable.

## Guarantor

The Guarantor for this article is John Cantanzaro, MD.

## Provenance and peer review

Not commissioned, externally peer-reviewed.
